# Human, All Too Human? An All-Around Appraisal of the “Artificial Intelligence Revolution” in Medical Imaging

**DOI:** 10.3389/fpsyg.2021.710982

**Published:** 2021-09-28

**Authors:** Francesca Coppola, Lorenzo Faggioni, Michela Gabelloni, Fabrizio De Vietro, Vincenzo Mendola, Arrigo Cattabriga, Maria Adriana Cocozza, Giulio Vara, Alberto Piccinino, Silvia Lo Monaco, Luigi Vincenzo Pastore, Margherita Mottola, Silvia Malavasi, Alessandro Bevilacqua, Emanuele Neri, Rita Golfieri

**Affiliations:** ^1^Department of Radiology, IRCCS Azienda Ospedaliero Universitaria di Bologna, Bologna, Italy; ^2^SIRM Foundation, Italian Society of Medical and Interventional Radiology, Milan, Italy; ^3^Academic Radiology, Department of Translational Research, University of Pisa, Pisa, Italy; ^4^Department of Computer Science and Engineering, University of Bologna, Bologna, Italy

**Keywords:** artificial intelligence, medical imaging, ethics, medico-legal issues, patient data, communication, psychology

## Abstract

Artificial intelligence (AI) has seen dramatic growth over the past decade, evolving from a niche super specialty computer application into a powerful tool which has revolutionized many areas of our professional and daily lives, and the potential of which seems to be still largely untapped. The field of medicine and medical imaging, as one of its various specialties, has gained considerable benefit from AI, including improved diagnostic accuracy and the possibility of predicting individual patient outcomes and options of more personalized treatment. It should be noted that this process can actively support the ongoing development of advanced, highly specific treatment strategies (e.g., target therapies for cancer patients) while enabling faster workflow and more efficient use of healthcare resources. The potential advantages of AI over conventional methods have made it attractive for physicians and other healthcare stakeholders, raising much interest in both the research and the industry communities. However, the fast development of AI has unveiled its potential for disrupting the work of healthcare professionals, spawning concerns among radiologists that, in the future, AI may outperform them, thus damaging their reputations or putting their jobs at risk. Furthermore, this development has raised relevant psychological, ethical, and medico-legal issues which need to be addressed for AI to be considered fully capable of patient management. The aim of this review is to provide a brief, hopefully exhaustive, overview of the state of the art of AI systems regarding medical imaging, with a special focus on how AI and the entire healthcare environment should be prepared to accomplish the goal of a more advanced human-centered world.

## Introduction

The term artificial intelligence (AI) was coined in 1956 to differentiate the intelligence of machines (generated by software development programs) from natural, human intelligence. In the past decade, AI algorithms have begun to influence many activities based on computer platforms, having a significant impact on daily life. These new technologies have aroused great interest among biomedical scientists, since AI has proven to be able to simplify the work of researchers and healthcare professionals, and to provide crucial information for the management of patients (e.g., early diagnosis, prediction of individual prognosis, and therapy personalization), which would realistically be very difficult or impossible to obtain without the support of such systems (Yu et al., 2018).

Radiology is one of the medical specialties with a greater interest in AI, since the latter can offer radiologists new tools for quantitative analysis and image interpretation in addition to offering automation and standardization of processes and procedures, which allow saving time and effort during fatiguing and/or repetitive tasks, improving diagnostic performance, and optimizing the overall workflow (Curtis et al., 2018; Hosny et al., 2018; Pesapane et al., 2018a; Yu et al., 2018; European Society of Radiology (ESR), 2019b; Grassi et al., 2019). However, this enthusiasm is paralleled by concerns of psychological, ethical, and medico-legal nature (including those related to the involvement of AI systems in patient management and the responsibilities that this may entail), as well as by the fear that AI could revolutionize radiologists’ jobs, possibly threatening their existence as specific professional figures (Gong et al., 2019; Savadjiev et al., 2019; van Hoek et al., 2019). More generally, it has been emphasized that as long as AI becomes more and more autonomous (e.g., able to talk, “think,” and actively participate in decision making), its role within complex relationships such as those between patients and physicians may be unclear to the human interlocutors, and new obstacles to decision making could arise due to AI acting as a “third wheel” between them (Triberti et al., 2020).

This review illustrates the main characteristics of AI systems and the current issues related to their use in the radiology profession.

## Artificial Intelligence: Basic Concepts

Broadly speaking, AI encompasses the ability of hardware and software devices to autonomously mimic activities which have traditionally been deemed as specific to humans, such as learning and thinking. More than 60 years after its inception, AI has recently come back under the spotlight owing to the increasing availability of relatively low-cost computers capable of processing large amounts of data in real time, enabling the practical implementation of AI systems. In the medical field, AI mainly refers to the ability of systems to detect and analyze data related to patient clinical information and management with the aim of accomplishing a predetermined goal (Savadjiev et al., 2019).

Artificial intelligence systems can be broadly classified into strong (or general) and weak (or restricted). Strong AI systems can apply AI to resolve any problem, and as such, aim to mimic human intelligence. Conversely, weak AI denotes systems from which humans can take advantage to efficiently perform specific tasks (Zackova, 2015; Brink et al., 2017; Park and Park, 2018; European Society of Radiology (ESR), 2019b). More specifically, weak AI systems can improve their intrinsic ability to solve problems autonomously by means of progressive learning, starting from acquired information. This category includes most systems used in practice which are based on different machine learning (ML) techniques, including those of bio-inspired artificial neural networks.

### Machine Learning

In 1959, Arthur Samuel gave a boost to the development of weak AI by introducing the concept of “ML,” defined as a subclass of AI systems which help the machine to learn and make decisions based on the data. To this end, the machine builds its own model from a subset of data used for training (Bishop, 2006; Litjens et al., 2017). Consequently, ML can make predictions on new data based on previous training without the need of being specifically programmed or recall previously defined models.

Another notable feature of ML is that the system performance increases with increasing experience of the system itself. In classic ML (which is used for classifying and interpreting data related to image analysis), data are labeled by human experts and organized according to their properties using statistical methods (Chartrand et al., 2017). In order for an ML algorithm to successfully reproduce the process of analyzing an image (e.g., a chest X-ray) by a radiologist, it must first be trained with a supervised approach starting from different labeled learning datasets (which contain many heterogeneous types of radiographic abnormalities), reinforced with different datasets each containing a class of abnormal findings (e.g., cardiac, mediastinal, pulmonary, and bone) and, if necessary, additionally reinforced with specific datasets for various subclasses of anomalies (e.g., congenital heart disease).

In general, ML is the highest expression of the power of a computer system. However, as in human learning, ML can also encounter some problems. For example, if the training dataset is poorly representative of the characteristics to be analyzed, an ML algorithm could learn from the training dataset in too much detail, leading to the problem of overfitting. In this case, non-significant statistical fluctuations of the same sample are cataloged by the learning model as separate data, which subsequently causes a worsening of the performance in analyzing new data (Duda et al., 2001; European Society of Radiology (ESR), 2019b). In diagnostic imaging, overfitting can be amplified by the possibility of non-pathological anatomical variants (such as accessory bones, or congenitally absent or hypoplastic structures).

The need for numerical accuracy and precision in processing radiological images represents one of the main challenges for the applications of ML systems in diagnostic imaging. Accuracy is essential for addressing the complexity of semantic aspects (related to the enormous variety of normal and pathological findings that an ML system could encounter in the analysis of images acquired on real patients) and technical issues due to differences among various imaging modalities. Another challenge is related to the large number of images which need to be processed from cross-sectional imaging modalities (even with the aid of semi-automatic algorithms), with magnetic resonance imaging (MRI) or multislice computed tomography (MSCT) being able to furnish hundreds or thousands of images per single dataset.

### Artificial Neural Networks and Deep Learning

**FIGURE 1:**
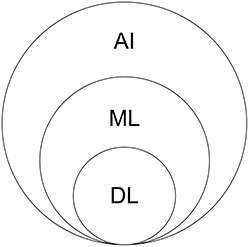
Schematic representation of the hierarchical relationship between AI (which, in general, describes systems capable of imitating human behavior), ML (a subset of AI, which describes systems capable of learning by experience), and DL (a subset of ML, which describes systems based on neural networks).

The term deep learning (DL) was first introduced in 1986 by Rina Dechter, and represents a form of ML which can yield better performance than classic ML (Figure 1). Compared to a traditional artificial neural network, in which the number of levels is limited and the nodes of one level are connected to those of the next level (“completely connected”), DL systems are generally made up of several specialized levels. The last levels are generally the only fully connected ones and combine functionalities learned to make decisions. Instead of requiring labeling or engineering of the properties, DL algorithms independently learn the most suitable characteristics for classifying the data provided, depending on the specific task (Chartrand et al., 2017; Philbrick et al., 2018).

The commonest approach in image processing is represented by convolutional neural networks (CNNs), a particular type of neural network developed for the recognition of patterns within images, which can accept two- or three-dimensional images as input (Domingos, 2012). While the first CNN was implemented in 1980 by Fukushima (1980), CNNs were formalized as we know them now by LeCun et al. (1998). The introduction of advanced graphic processing units with the ability to process enormous amounts of data in parallel has made CNNs an essential tool for the development of modern DL algorithms (Hinton and Salakhutdinov, 2006; Trebeschi et al., 2017).

The two factors which mainly affect the functionality of CNNs are the power of the hardware and, most importantly, the availability of adequate data for the learning process. If computer power increases progressively over years or months, and can therefore only be relatively limiting, time and cost constraints make it difficult to find a solution to the problem of the low availability of well-structured datasets for training, which represents an actual hurdle to the development and diffusion of these systems (Napel et al., 2018).

Deep learning has proven to be a promising tool for the extraction of features from biomedical images (LeCun et al., 2015; Wang et al., 2017; Kermany et al., 2018; Lustberg et al., 2018). For that application, computational units are defined as levels integrated with each other to extract the intrinsic characteristics of the images. Using a CNN structured in a hierarchical manner, a DL system can, for example, extract the intrinsic characteristics of a neoplasm to build a model capable of providing prognostic or predictive information, having a clear potential impact on patient clinical management (Wang et al., 2019).

### Artificial Intelligence in Medical Imaging: “Images Are More Than Pictures, They Are Data” (Gillies et al., 2016)

During its development, medical imaging has enjoyed great benefit from technological progress (Nance et al., 2013; Nguyen and Shetty, 2018), and the scientific relevance of the development of AI systems in radiology has been underscored by an ever-increasing number of publications on AI. For diagnostic imaging alone, the number of publications on AI has increased from about 100–150 per year in 2007–2008 to 1000–1100 per year in 2017–2018 (Tang, 2020).

Artificial intelligence systems can support medical decision-making processes related to requests for imaging tests, not only by means of the evaluation of the patient’s medical record and the accuracy of the radiological examinations, but also by guiding the choice of the most suitable diagnostic modality. Of note, AI algorithms can be programmed to work in keeping with the appropriateness criteria developed and approved by scientific societies (such as those developed by the American College of Radiology) in order to maximize the adherence to validated criteria (Blackmore and Medina, 2006; American College of Radiology (ACR), 2021 Reporting and Data Systems).

Furthermore, AI has opened new perspectives on how to make the most of the information which can be obtained from biomedical imaging for a more in-depth understanding of the various pathological processes, aimed at more effective diagnostic and therapeutic management. Once trained with appropriate learning datasets, AI systems can analyze biomedical images with the aim of recognizing specific characteristics (either visible or invisible to the human eye) and build probabilistic models capable of detecting abnormal findings (Dodd, 2007; Sardanelli et al., 2010).

**FIGURE 2:**
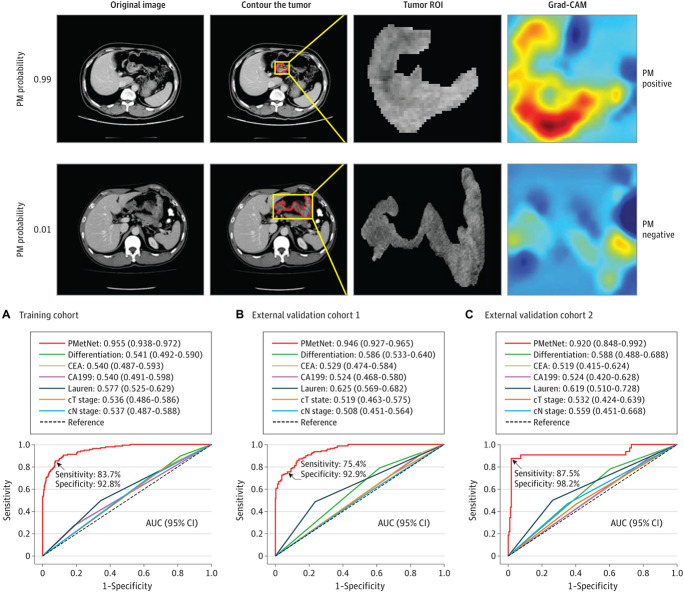
Evaluation of a CNN-based DL algorithm (PMetNet) for detection of occult peritoneal metastases (PM) from gastric cancer on preoperative CT images. (Upper) Following tumor segmentation on original CT images (tumor contours highlighted in red, tumor region of interest [ROI] shown within magnified tumor location [boxed in yellow]), the gradient-weighted class activation mapping (Grad-CAM) was used to highlight which areas of the CT image are important in generating a particular prediction. The numbers 0.99 and 0.01 represent the predicted probability of PM. (Lower) Area under the receiver operating characteristic curves (AUCs) derived from the PMetNet and clinicopathological factors for the diagnosis of occult PM in the training cohort and in two validation cohort. The discrimination performance of PMetNet was substantially higher than conventional clinicopathological factors, and in multivariable logistic regression analysis, PMetNet was an independent predictor of occult PM. Adapted from Jiang et al. (2021) under Creative Commons Attribution 4.0 (CC BY 4.0) license (https://creativecommons.org/licenses/by/4.0/).

Automated image interpretation is one of the potential radiological applications of AI which has been received with the greatest enthusiasm. Rajpurkar et al. (2018) illustrated an AI algorithm with a comparable accuracy to that of human radiologists for diagnosing pneumonia on chest X-rays in a public dataset. Similar experiences have been reported for the detection of vertebral fractures on plain spinal radiography (Murata et al., 2020), the diagnosis of tuberculosis (Lakhani and Sundaram, 2017), and the estimation of bone age (Dedouit et al., 2015). More generally, different DL methods have been applied to biomedical image analysis (Ierardi et al., 2016; Trimboli et al., 2018; Villanueva-Meyer et al., 2019) and successfully used with various imaging modalities, such as breast (Kallenberg et al., 2016; Zanotel et al., 2018; Geras et al., 2019; Hickman et al., 2021) and cardiac imaging (van Assen et al., 2020), MSCT (Lerouge et al., 2015; Kooi et al., 2017; Hu et al., 2020), MRI (Havaei et al., 2017; Ariji et al., 2019; Figure 2), as well as in interventional radiology (Gurgitano et al., 2021). AI can also be helpful to quantify lung involvement and predict prognosis in patients with COVID-19 pneumonia (Belfiore et al., 2020; Akram et al., 2021; Cappabianca et al., 2021), and Harmon et al. (2020) recently found that a series of DL algorithms trained in a diverse multinational cohort of 1280 patients can achieve up to 90.8% accuracy, with 84% sensitivity and 93% specificity in detecting COVID-19 pneumonia on chest CT examinations of 1337 patients. Other AI applications allow prioritizing the reporting of certain exams (e.g., urgent brain CT scans in patients with hemorrhagic stroke), thus optimizing the workflow and avoiding diagnostic delays, especially in situations in which members of the radiology department are busy with other tasks (Ngo et al., 2017; European Society of Radiology (ESR), 2019b). However, there are currently no commercial solutions available which can independently interpret images and generate a report.

The ability of extracting structured and categorized data from existing radiological archives [radiology information system (RIS) and picture archiving and communication system (PACS)] is an essential requirement for the development and dissemination of AI in radiological environments. In fact, the training of AI systems usually requires enormous amounts of data which should be as accurate and correctly categorized as possible. However, to date, most radiological reports are written in the form of an unstructured narrative text which greatly complicates the extraction of information, even if the aim is to create AI systems based exclusively on clinical data. The latter could be resolved with the adoption of structured reporting (SR), which, if properly implemented, would allow the exchange of information with a common lexicon and semantics. While there is extensive evidence that SR has several advantages over conventional unstructured reporting (including better clarity, improved communication with patients and referring physicians, higher productivity, and ease of data mining), it has seen a relatively slow diffusion so far due to radiologists’ fears, among others, that it could diminish their autonomy and professional reputation with respect to patients or non-radiologist specialists (Marcovici and Taylor, 2014; Faggioni et al., 2017; Coppola et al., 2021). In this context, constructive interaction between medical radiologists and other specialists, industries, and institutions would be desirable to promote a large-scale dissemination of the SR, offering a decisive stimulus for additional development of AI in the radiological field (Bosmans et al., 2015; Pinto Dos Santos and Baeßler, 2018; European Society of Radiology (ESR), 2019b).

A topic of interest for both the biomedical industry and research is the prospect of using AI to optimize biomedical image acquisition protocols, with potential advantages in terms of patient safety and health management costs. For example, some AI algorithms allow obtaining equivalent or even superior results as compared to commercial non-AI-based solutions for noise reduction in MSCT and positron emission tomography (PET) examinations, allowing the acquisition of diagnostic images with a significantly lower radiation exposure than conventional protocols (Zhu et al., 2018; Shan et al., 2019).

Radiomics is a field of research which has become very popular in the era of modern precision medicine. Radiomics refers to the established use of ML techniques applied to the analysis of radiological images. Radiomics is often defined as “the extraction of a large number of quantitative features from conventional biomedical images in order to obtain data that can be used in clinical decision support systems to improve diagnostic, prognostic, and predictive accuracy” (Choy et al., 2018). A radiomic model can reveal the value of biomarkers extracted from the images (which are quantifiable by means of applying mathematical and statistical models, even of considerable complexity), but it can also extend to a so-called hybrid model, including other data not from images (e.g., from clinical data or laboratory parameters). In any case, these models are able to provide information not obtainable with standard radiological semeiotics, such as those related to the early response to treatment, the prediction of the biological aggressiveness of a neoplasm, the existence of molecular targets for any targeted therapies, up to the prediction of the individual prognosis, and the personalization of therapies (Gillies et al., 2016; Lambin et al., 2017; Choy et al., 2018; Bi et al., 2019; Liu et al., 2019; Rogers et al., 2020; Zerunian et al., 2021). A notable feature of radiomics consists of the possibility of obtaining, in a repeatable and non-invasive way, information about a tissue in its entirety in contrast, for example, to what happens with a classic biopsy which is invasive and limited to a portion of tissue, with the risk of collecting a sample that is not representative of the heterogeneity of the lesion (Lambin et al., 2017; Abdollahi et al., 2019; Nazari et al., 2020; Zerunian et al., 2021). The radiomics approach can also be extended to the analysis of the genetic structure of a tissue (e.g., neoplastic), which is referred to as radiogenomics (King et al., 2013; Pinker et al., 2018; Story and Durante, 2018; Gabelloni et al., 2019; Lo Gullo et al., 2020). A radiomic biomarker is made up of a set of characteristics (or features) extracted from the image, it is represented by a mathematical equation, known as a “signature,” and its value can be calculated using dedicated programs, starting from images acquired using routine protocols.

However, even if many of these tools are easy for operators to use and allow extracting radiomic features in a relatively short time, their diffusion is currently still limited by several factors. These include the enormous amount of work often necessary for image segmentation and the difficulty in ensuring the adequate quality of the data entered to obtain consistent results, also considering the inevitable differences both between the image acquisition protocols, and different machines and imaging centers (Stoyanova et al., 2016).

## Artificial Intelligence and Humans

Several authors have hypothesized that AI systems might shortly be able to replace medical radiologists in their professional activity (Rizzo et al., 2018). The key question is: will AI be able to replace radiologists in the observation, characterization, and quantification tasks that they currently accomplish using their cognitive skills? (Beregi et al., 2018; Tajmir and Alkasab, 2018; Mendelson, 2019; Miller and Brown, 2019; Rubin, 2019). The short answer is: NO. However, as argued by Dr. Curtis Langlotz at the European Congress of Radiology in 2018: “AI won’t replace radiologists, but radiologists who use AI will replace radiologists who don’t” (Krittanawong, 2018), and this concept could be generalized to all fields of healthcare (Meskó et al., 2018). In this context, it is important to point out that the final decision regarding patient diagnosis is still autonomous and the responsibility lies with the radiologist, not AI systems. What is most likely to change will be the use of information not only derived from morphological analysis in the formulation of the diagnosis, but also from the numerical values provided by AI. These refer directly to statistically significant distributions of the pixel values of the image which are not perceptible to the naked eye.

Both radiologists and AI systems must follow essential rules and principles for optimal patient management. Several issues are related to the proper use of AI in clinical practice and include (but are not limited to) the following:

• Data (including generation, recording, maintenance, processing, dissemination, sharing, and use)
• AI algorithms used to process patients’ data for a specific task
• Practices (including responsible innovation, programming, security, formulation, and implementation of ethical solutions)
• Communication (including the tools through which the information obtained from AI systems is provided to patients, as well as the management of psychological problems arising from them, which cannot be handed over to a computer system) (Meskó et al., 2018).

Various aspects of data ethics can be recognized, including informed consent, privacy and data protection, data ownership, objectivity in managing data, and the likelihood that a gap may exist between those who have the resources to manage and analyze large amounts of data and those who do not. In addition, the operation of AI systems integrated into big data networks raises ethical and legal issues related to patient-specific consent, data sharing, privacy and security protection, and the availability of multi-layered access to fully or partially anonymized health information.

### Artificial Intelligence Overconfidence and Medico-Legal Issues

As stated by Kulikowski (2019), “whether a good ethical human can work with an AI and remain ethical is a major open problem for all of us that will have to be confronted not only scientifically, but also in a socially acceptable and humanistic way in clinical informatics.” Hence, ethics should always guide radiologists (and physicians in general) in deciding when to rely on AI, so as to avoid improper applications of it which may have a harmful impact on both healthcare operators and patients.

One of the main biases which can hamper the use of AI in diagnostic imaging is the automation bias, which can be defined as the propensity to favor a machine-generated diagnosis over evidence derived from scientific knowledge and the physician’s own expertise. This leads to the so-called omission and commission errors. Omission errors occur when the physician, deeming AI flawless, does not notice (or outright ignores) the fallacy of one of its tools. On the other hand, commission errors occur when a machine’s decision is accepted, even in the face of contrary evidence. The risks of automation bias can be amplified in realities which suffer from a lack of medical personnel, since there may not be any radiologist to double-check the AI results (Geis et al., 2019). It has also been observed that automation could engender overreliance by its users (due to its advantages in terms of increased efficiency), and in the long term, lead to the so-called deskilling, with physicians losing their ability to autonomously perform tasks which have become automated (Cabitza et al., 2017). Harada et al. (2021) performed a randomized controlled study aimed to explore the prevalence of AI diagnoses in physicians’ differential diagnoses when using an AI-driven diagnostic decision support system (DDSS) based on the information entered by the patient before the clinical encounter, showing that at least 15% of physicians’ differential diagnoses were affected by the differential diagnosis list in the AI-driven DDSS. While many clinicians hope that AI will free them to focus on patient interaction, research on the overreliance of technology in medicine has found that the increased use of electronic health records has led to a prioritization of physician–technology interactions over physician–patient interactions, leading to decreased patient satisfaction, a scenario that could foreshadow the role of AI in patient care (Lu, 2016; Ross and Spates, 2020).

There is a still highly unmet need for specific guidelines, policies, and recommendations offering an ethical framework that can guide the use and implementation of AI technologies in an increasingly broad spectrum of clinical applications, which are progressively emerging as an effect of technological evolution, but also carry substantial psychological and ethical implications. Some of such potential applications include, for instance, AI in assisted reproductive technologies for human embryo selection *in vitro* fertilization (Dirvanauskas et al., 2019), and optimization of clinical trials of innovative stem cell and gene therapies in pediatric patients by precise planning of treatments, simplifying patient recruitment and retention, and lowering their complexity and costs (Sniecinski and Seghatchian, 2018). However, despite efforts by scientists, healthcare professionals, administrative managers, and lawmakers, so far very few countries worldwide have adequate and critical governance frames allowing best understanding and steering AI innovation trajectories in healthcare (Dzobo et al., 2020).

Such scenario is further complicated by the sweeping speed at which AI techniques are being developed or sometimes used, even before the publication of appropriate policies and guidelines, which might leave users confused about how to best integrate this new technology into their practice. This implies that updated regulatory policies and continuing education of all users (including adequate information to patients about the purposes, rights, and legal terms related to the use of AI for their health management) should be promoted, as AI systems are poised to become more widely available, complex and powerful. To this purpose, it is noteworthy that the majority of Singaporean radiology residents joining a national multiprogram survey thought that since AI will drastically change radiology practice, AI/ML knowledge should be taught during residency (84.8% of survey participants), and this was as important as imaging physics and clinical skills/knowledge curricula (80.0 and 72.8%, respectively) (Ooi et al., 2021). From a psychological standpoint, it has been observed that openness to experience is associated with higher trust toward robots and AI, as well as having a degree in technology or engineering, exposure to robots online, and robot use self-efficacy (Oksanen et al., 2020), highlighting the importance of technology knowledge in addition to personal differences in building AI confidence.

**FIGURE 3:**
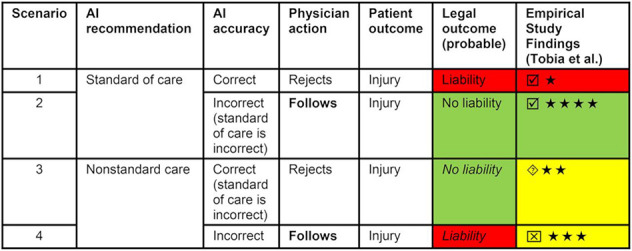
Comparison of potential legal outcomes under current law according to analysis of Price et al. (2019) and empiric study findings of Tobia et al. (2021). ★ = agreement that physician decision was reasonable (highest is ★★★★; lowest is ★). Greater agreement indicates lower likelihood of liability; ☑ = study results confirming Price et al.’s analysis of current tort law; 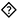 study results suggesting that jury outcome may also be liability; 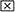 study results suggesting that jury might decide no liability. Reproduced from Price et al. (2021). © SNMMI.

A key medico-legal aspect regarding the use of AI in healthcare is the responsibility for the decision-making processes upon which the patient’s health depends. In the absence of specific regulations, there may be ethical and medico-legal issues where an AI system is involved in the process and may suggest solutions (right or wrong); however, the final decision (also right or wrong) is, and will always be, the responsibility of the physician who is legally responsible (Mittelstadt and Floridi, 2016; Price et al., 2019, 2021; Reddy et al., 2019; Neri et al., 2020). Price et al. (2019, 2021) provided an in-depth analysis of the potential legal outcomes related to the use of AI in healthcare under current law (Figure 3).

Another problem is the opacity related to AI models being mostly a “black box” without a universal understanding of their inner workings, which are not acceptable for decision support solutions (especially in healthcare) and may lead to ethical and legal risks and liability issues, as well as undermine patients’ and physicians’ confidence into AI (Valiuškaitė et al., 2020; Tohka and van Gils, 2021). Putting this issue in the context of radiological practice, radiologists would be asked to monitor AI system outputs and validate AI interpretations, so they would risk carrying the ultimate responsibility of validating something they cannot understand (Neri et al., 2020). There is a clear difference between statistical and clinical validation, and hence achieving adequate informed consent is problematic when the algorithmic decision-making process is opaque to clinicians, patients, or courts (Martinez-Martin et al., 2018; Arnold, 2021). Actually, while the number of published articles on the applications of AI in medical imaging and other medical specialties is steadily increasing, so far only few AI applications have been validated for clinical use, partly due to the difficulty of using AI projects on a large scale in real-life clinical practice, poor adherence to scientific quality standards (Nagendran et al., 2020; Park et al., 2020), and clinical validation issues. While the ongoing development of AI has generated considerable hype and highly optimistic expectations in the scientific community, such enthusiasm is often curbed by the reality of proper performance assessment, which is not trivial (requiring an understanding of the problem and data) and is often costly (data needs to be reserved) and time consuming (Tohka and van Gils, 2021). According to some researchers, the overall problem is wide and ultimately originates from inappropriate experimental design and hypothesis testing procedures, including so-called Hypothesizing After the Results are Known (aka HARKing) practices (Gencoglu et al., 2019; Tohka and van Gils, 2021).

The process of technology and infrastructure development requires close multidisciplinary cooperation among governmental institutions, research centers, healthcare professionals, and industry. In this context, a potential solution could involve enrolling AI experts in radiology units to act as a link between AI systems and radiologists who, in turn, should be trained to use those systems independently. To ensure that the approach to an innovative and potentially destructive technology is properly managed, radiologists will have to develop strategies not based on prejudice, but specifically adapted to the peculiar characteristics of AI systems, their technical/scientific development, their implementation by the industry, and their actual diffusion.

### Data Confidentiality and Regulation Policies

Using AI systems for diagnosing diseases (including life-threatening or invalidating diseases, with a potentially dramatic impact on the physical and psychological well-being of patients and their families) and finding the most appropriate therapeutic approach implies that these systems should have the highest grade of reliability and dependability. To this end, the following requirements should be met:

• The largest possible amount of data (both imaging and non-imaging-related) should be shared.
• The quality and integrity of these data should be as high as possible, avoiding errors due to poor image quality, mislabeling, and over- and underfitting.
• The anonymity and depersonalization of data must be guaranteed so as to ensure that the individual(s) who has/have consented to their use can be traced.

In recent years, several authors have discussed the requisites for the reliable innovation of AI by means of attaining the most important ethical principles. These principles have been embodied in the laws of the various countries throughout the world, and while an overarching political vision and long-term strategy for the development of a “good AI” society are currently lacking, this process has been characterized in broad terms by the search for a tradeoff between AI technological innovation and regulation (Cath et al., 2018; Pesapane et al., 2018b; Monreale, 2020).

As mentioned by Cath et al. (2018), the political attitude of the United States toward the implementation of AI in healthcare can be summarized by the sentence: “Letting a thousand flowers bloom,” whereas that of the European Union (EU) can be described as: “European standards for robotics and AI,” and the UK approach (“Keep calm and commission on”) stands in an approximately intermediate position between the US and the EU policies. In the United States, a “Silicon Valley” model oriented toward the more liberal regulation of ethical issues and based on the “move fast, break things first, apologize later” approach has prevailed (Armitage et al., 2017) and, in 2020, the Trump administration published a guide for AI application which discouraged any action resulting in limiting innovation and technological progress (Vought, 2020). At the other extreme, the EU policy points to strictly codifying regulations based on ethical principles. While this policy has raised the objection that it could hinder AI innovation, the European Commission sees the codification of ethical principles for AI use as a competitive advantage which will promote consumer confidence in their products and harmonize their adoption across the EU (Monreale, 2020). Data protection in the EU is regulated by General Data Protection Regulation (GDPR) EU 2016/679 and other EU directives for confidential data protection, which is of paramount importance in case of the AI/ML systems being trained on personal healthcare data (Voigt and Von dem Bussche, 2017). In this respect, the principles of “privacy by design” described by Cavoukian (2009) and updated by Monreale et al. (2014) could be applied, in the perspective of promoting research and innovation while taking care and full responsibility of the protection of the personal data, rights, and freedom of EU citizens.

It is especially important that the requirements for privacy protection are fulfilled during the process of data extraction for the training of ML algorithms, avoiding the potential risks related to illegal access either to confidential data during training or to the ML model used for clinical patient management (Brundage et al., 2018; Pesapane et al., 2018b; Monreale, 2020). On the other hand, current data protection laws may pose a significant limitation for researchers who develop and use ML algorithms, resulting in a lack of generalization of training which has so far prevented a more widespread application of such algorithms into clinical practice by healthcare providers across the world. This holds especially true for rare diseases, for which the accuracy of ML algorithms could be limited due to the relatively small amount of data for algorithms to train on and data collection is inherently slow due to a low disease prevalence. Similarly, algorithms which predict outcomes from genetic findings may lack generalizability if there are only a limited number of studies in certain populations (Kelly et al., 2019; Abayomi-Alli et al., 2021; Tohka and van Gils, 2021).

Machine learning models are programmed based on de-identified data, i.e., those which do not directly allow identifying an individual in a univocal manner. Unfortunately, in some contexts, de-identification is not sufficient to protect a person’s privacy, since individuals could be indirectly reidentified by means of the correlation of the de-identified data with public data (Jones, 2018), prompting the adoption of more advanced solutions aimed at fully protecting patient anonymity, such as k-anonymity (Sweeney, 2002). From a more general ethical and legal viewpoint, while patient data stored in electronic health records may be de-identified and, through data linkage, generate beneficial research outcomes, this may create a tension between beneficence (for the public) and private confidentiality, overriding contemporary notions of privacy and confidentiality according to the duty of “easy rescue,” particularly in circumstances of minimal risk as defined by research regulators (Porsdam Mann et al., 2016; Arnold, 2021). Moreover, a study from the University of California, Berkeley, suggests that progress in AI has rendered the privacy standards set by the Health Insurance Portability and Accountability Act of 1996 (HIPAA) obsolete (Na et al., 2018). The important conclusion is that privacy standards associated with the current legal and regulatory framework should be revisited and reworked, such that the advances of AI and its impact on data privacy as it pertains to healthcare are factored in (Na et al., 2018; Ahuja, 2019; Kulkarni, 2021).

An additional risk regarding a breach of patient confidentiality could derive from so-called “membership inference attacks,” i.e., malicious attacks toward AI algorithms which are aimed at detecting the confidential data used to build the algorithm (Shokri et al., 2017). Actually, the implementation of AI systems means access to sensitive health data, which intrinsically always carries the risk of cyberattacks, posing a substantial risk on the privacy of patients (especially those with lower education and financial income; Bilen and Özer, 2021) and requiring a guaranteed level of robustness against such attacks (Catak et al., 2021; Zhou et al., 2021). Attacks on AI systems can undermine diagnostic accuracy, administer lethal drug doses, or sabotage critical moves in an operation, and in the area of diagnostic imaging, they can manipulate data entering AI systems (so-called “input attacks”), leading to false diagnosis and altered patient care and/or reimbursement (Finlayson et al., 2019; Kiener, 2020; Myers et al., 2020). The malware can obtain personal information by means of query and repersonalization of the data within the algorithm, and most strategies aimed at offering protection against such privacy violations rely on methods based on differential privacy, i.e., a privacy model based on the concept of data perturbation (Bugliesi et al., 2006). While several solutions have been proposed to forecast, prevent, and mitigate threats from malicious uses of AI technologies, a coordinated action of all involved stakeholders (including researchers, engineers, and AI users) has been advocated to manage what is expected to become a long-term equilibrium between AI attackers and defenders (Brundage et al., 2018).

### Accessibility of Artificial Intelligence Services

An important issue that should receive special attention is related to the possibility that the access to AI systems may be not equal for all patients or healthcare professionals. In fact, smaller facilities and academic centers with fewer resources may lack the means to acquire (and skills to use) complex and more performing AI systems. Furthermore, if AI were to be developed and exclusively owned by large entities in the private sector, this would likely further restrict its spread to a wider public. Collaboration between academic institutions and the public and private sectors has been advocated to foster the development of both workforce and AI applications in healthcare (Mikhaylov et al., 2018; Ishii et al., 2020).

The so-called “digital divide” can be classified as global, social, and democratic (Srinuan and Bohlin, 2011); in any case, it invariably implies that affected subjects are excluded from the benefits of technological progress and innovation. A realist review in general practice by Huxley et al. (2015) showed that while digital communication technology offers increased opportunities for marginalized groups to access health care, it cannot remove all barriers to care for these groups, and actually they will likely remain disadvantaged relative to other population groups after their introduction. There is a risk that such phenomenon may occur in a previously unseen fashion with AI, with factors including age, gender, health condition, level of education, or financial income possibly leading to unequal access to AI systems.

An increasingly recognized issue is the potential for bias of AI systems with respect to certain population subgroups with a lack of diversity (e.g., in age, ethnicity, socioeconomic background, etc.) if algorithms have been developed on datasets which under- or over-represent them (Caliskan et al., 2017; Reddy et al., 2019; DeCamp and Lindvall, 2020; Hickman et al., 2021). Algorithmic bias may occur in ML systems for healthcare, perhaps predicting a greater likelihood of disease on the basis of gender or race when those are not actual causal factors (Davenport and Kalakota, 2019). Other issues could arise from discriminatory behaviors toward socially weaker individuals, from the need to gain the physicians and patients’ trust in a context where AI systems process biomedical data and play a crucial role in clinical management, or from the duty of providing concrete rights of access to services to each patient. The communication of medical information, rules for the use of data, and requirements for institutional review committees may need to include new possibilities for patient data management (Kohli and Geis, 2018).

### Communication and Psychological Issues

As outlined previously, while AI is supposed to offer radiologists substantial aid in their professional activity, predictions that it will replace radiologists in a more or less distant future are unfounded since the professional role of radiologists involves many tasks which cannot be accomplished by computers alone, including carrying out interventional radiology procedures, performing a clinical-radiological correlation in image interpretation, interpreting complex findings, and communicating them to colleagues and/or patients (Russell and Bohannon, 2015; Price et al., 2019). However, radiologists will have to improve their relationship with patients in the AI era to avoid any patient discomfort due to a lack of empathy and of a human reference figure during all the steps of a radiological procedure. These range from the patient’s admission to the communication and discussion of imaging findings (the latter being a source of considerable psychological stress for patients and, hence, a task which could not be assigned to any, however perfect, AI algorithm). Moreover, reaching a diagnosis may often involve the use of multiple imaging techniques which are proposed by the radiologist (in combination with clinical and laboratory data, as well as with other non-radiological tests), and the overall interpretation of imaging findings is a complex task which requires a global assessment of the patient’s condition and as such cannot be demanded to a computer system. In this context, the ability of AI systems to not only improve the detection and characterization of diseases (e.g., cancer) but also guide treatment and predict individual patient outcomes and prognosis (Bi et al., 2019; Rogers et al., 2020) can create additional issues related to the complexity of communicating and discussing topics with a high emotional impact (Butow and Hoque, 2020).

While AI allows saving time regarding the diagnostic and therapeutic decision process, the latter could actually be delayed if the role of AI is not taken into account in the consultation between physician and patient (Pravettoni and Triberti, 2019) (who may wish, and has the right to, know the implications of using AI in his/her clinical management), or if AI conclusions need to be revised by human doctors (especially when important decisions are to be made based on such conclusions) (Triberti et al., 2020). Moreover, as mentioned above, the poor explainability of most current AI systems (which are undoubtedly characterized by a high degree of complexity) and their lack of transparency could engender anxiety, distrust, or outright hostility with respect to AI in patients and clinicians (Bi et al., 2019). The relationship between patient and physician is a complex and profound psychosocial interaction characterized by mutual knowledge, trust, loyalty, and regard, so that human interaction will remain essential for patient-centered care due to the uniqueness of “human touch,” consisting of peculiar features (such as empathy or the ability to be in tune with other people’s thoughts and feelings) (Honavar, 2018). To this regard, it is known that a better communication between patients and physicians is associated with lower patient anxiety, fewer malpractice claims, and improved quality of life (Levinson et al., 2010). As to patients’ trust in AI performance, Juravle et al. (2020) reported three online experiments showing that given the option of receiving their diagnosis from AI or human physicians, patients trusted those latter more for both first diagnoses and a second opinion for high risk diseases, and their trust in AI did not increase when they were told that AI outperformed the human doctor, but the trust in AI diagnosis increased significantly when participants could choose their doctor.

Owing to their pivotal role in the diagnostic process, radiologists are often the first healthcare professionals who are asked by patients about their imaging findings (and hence find themselves to deal with patients’ emotional reaction), and from whom patients expect a direct communication of their imaging findings (Berlin, 2007; Capaccio et al., 2010; Cox and Graham, 2020). The use of AI systems with the ability to provide additional information which may have a significant impact on patient management and overall life (e.g., eligibility to specific treatment options, prognosis, etc.) will entail for radiologists more stringent requirements in terms of communication skills and psychological balance, as well as a high degree of constructive interaction and feedback with other medical and non-medical specialists involved in patient care.

A detailed knowledge of the main features of AI (including its technical background, its current fields of application, and its psychological and legal implications) is a preliminary condition for its usage as a mature professional tool (Kobayashi et al., 2019; Savadjiev et al., 2019; Sogani et al., 2020). In a nationwide online survey among members of the Italian Society of Medical and Interventional Radiology (SIRM), most radiologists (77%) were favorable to the adoption of AI in their working practice, with a lower diagnostic error rate and work optimization being main perceived advantages, whereas the risk of a poorer professional reputation compared with non-radiologists was seen as one major downside (60% of survey respondents). However, about 90% of surveyed radiologists were not afraid of losing their job due to AI, and less than 20% of them were concerned that computers will replace radiologists for reporting of imaging examinations (Coppola et al., 2021). To this respect, it is worth mentioning that while most medical students surveyed by Gong et al. (2019) were discouraged from considering the radiology specialty out of anxiety that AI could potentially displace radiologists, in that same study prior significant exposure to radiology and high confidence in AI understanding were associated with a lower anxiety level, suggesting that professional education can have a significant impact on the psychological attitude of physicians toward AI.

Moreover, in the aforementioned SIRM survey and in a EuroAIM survey aimed at assessing the perceived impact of AI in radiology among European Society of Radiology (ESR) members, most respondents believed that if AI systems will allow radiologists to save time, such time should be used to interact with other clinicians or patients, thus improving personal interaction and communication (European Society of Radiology (ESR), 2019a; Coppola et al., 2021). Similar findings were reported in a French survey including 70 radiology residents and 200 senior radiologists, whose main expectations about AI included a lower risk of imaging-related medical errors and an increase in the time spent with patients (Waymel et al., 2019).

In light of the above, AI could alleviate radiologists’ traditional work burden by undertaking tasks that could better be performed by computers, while giving them the opportunity to invest time and resources for other tasks that are better or uniquely accomplished by humans, such as interpreting imaging findings in the full width and complexity of a real clinical context, enhancing communication with patients and clinicians, supervising the correct operation and usage of AI systems, and being actively engaged in research (including AI-assisted data mining for big data handling and management of large-scale clinical trials) and quality optimization of the whole healthcare process. Like pathologists (who also extract medical information from images), radiologists will have an inescapable opportunity to leave once for all the stigma of “invisibility” which has often overshadowed the perception of their professional role by patients and clinicians in the past (Glazer and Ruiz-Wibbelsmann, 2011), and to take on a pivotal role in patient care as information specialists, adapting incrementally to AI and retaining their own services for cognitively challenging tasks and interaction with patients and clinicians (Jha and Topol, 2016; Recht and Bryan, 2017). Likewise, also clinicians need not fear AI as a potential enemy who could harm their professional reputation in the patients’ eyes or their jobs in the future, but they should leverage its power to tackle computationally and labor-intensive tasks better than humans and to concentrate on those tasks which require human action (Ahuja, 2019). Therefore, an enhanced professional role could be envisaged for both radiologists and clinicians, requiring more advanced and specific skills (Recht and Bryan, 2017; Krittanawong, 2018; Ahuja, 2019; Waymel et al., 2019), despite fears that AI taking over professional tasks once performed by humans could, in the long run, lead to deskilling of human physicians (Bisschops et al., 2019; Campbell et al., 2020; Panesar et al., 2020). AI could actually help radiologists and clinicians make the most of their own specialty knowledge and competence in a medical science of rapidly increasing complexity (where “diseases do not respect boundaries” between medical specialties and require the cooperation of multiple specialists; Deo, 2021), avoiding misunderstandings and “turf wars” due to poor communication and confusion regarding their specialty-specific roles in patient management, and possibly fostering the adoption of AI-augmented multidisciplinary teams (including software engineers and data scientists among participants) for clinical decision making (Di Ieva, 2019; Lee and Lee, 2020; Martín-Noguerol et al., 2021).

Other potential issues of AI in the physician–patient relationship include misunderstanding (since a disagreement between the physician and AI can cause confusion, and the patient may not recognize who has the real authority in the care management) and alienation due to the physician or patient feeling excluded from the contribution of AI. To this regard, it should be considered that AI is deficient in emotional intelligence, whereas a physician has skills, beliefs, and subjective perceptions which can shape the communication with the patient, thus seeking an adequate patient’s understanding of the disease and its related treatment options as the main aim of the communication process (Oh et al., 2017; Keskinbora, 2019; Pravettoni and Triberti, 2019; Triberti et al., 2020).

It has been observed that once digital and objective data will have become accessible to both caregivers and patients, the so-called “digital health” (of which AI is a major component) will lead to an equal level of physician–patient relationship with shared decision-making and a democratization of care (Meskó et al., 2017). However, it is possible that while some patients could accept or even require AI as an additional tool for decision making in their own medical care, others would not accept its use in decision-making (Meskó et al., 2018), thus stressing the need for setting out shared policies aimed to a rational utilization of AI in patient management. A recent study on patients’ perception about the use of AI for skin cancer screening as assessed by means of a semistructured interview revealed that most of them were favorable to AI and believed that it may improve the quality of care, but only if implemented in a manner which preserves the integrity of the human physician–patient relationship (Nelson et al., 2020). Again, direct physician–patient communication must be considered as an integral part of care delivery which cannot be substituted by a machine, and as Krittanawong has pointed out: “*AI cannot engage in high-level conversation or interaction with patients to gain their trust, reassure them, or express empathy, all important parts of the doctor–patient relationship*” (Krittanawong, 2018).

### eXplainable Artificial Intelligence and Causability: Forthcoming Steps for Artificial Intelligence to Enter Maturity?

In conclusion, while it is undeniable that AI will sooner or later affect healthcare and the professional role and work of healthcare providers, physicians should neither uncritically accept nor unreasonably resist developments in AI, but they must actively engage and contribute to an iterative discourse to preserve humanitarian concerns in future models of care (Arnold, 2021). In this context, it is clear that the sustainable use of AI involves keeping in mind its fields of applicability and limitations, thus envisaging a future where its capabilities and advantages integrate (rather than supplant) human intelligence.

The main future goal is to make AI capable of interacting with operators in a meaningful and easily accessible manner. In this context, eXplainable Artificial Intelligence (xAI) has emerged as a new discipline which tries to fulfill the need for causability in the medical domain; in the same way that usability encompasses measurements for the quality of use, causability encompasses measurements for the quality of explanations produced by xAI. Multi-modal causability is especially important in the medical domain, since results are often achieved by means of multiple different modalities. The key for future human–AI interfaces is to map explainability with causability, and to allow a domain expert to ask questions so as to understand why AI has come up with a result, and also to ask “what-if” questions (counterfactuals) to gain insight into the underlying independent explanatory factors of a result (Holzinger, 2021).

## Author Contributions

FC, LF, and MG: conceptualization. MG, GV, AP, and SLM: methodology. FDV, VM, AC, MAC, and LVP: literature review. FC, LF, FDV, and VM: writing–original draft preparation. MG, MM, SM, and AB: writing–review and editing. EN and RG: supervision and guarantee of scientific integrity. All authors have read and agreed to the published version of the manuscript.

## Conflict of Interest

The authors declare that the research was conducted in the absence of any commercial or financial relationships that could be construed as a potential conflict of interest.

## Publisher’s Note

All claims expressed in this article are solely those of the authors and do not necessarily represent those of their affiliated organizations, or those of the publisher, the editors and the reviewers. Any product that may be evaluated in this article, or claim that may be made by its manufacturer, is not guaranteed or endorsed by the publisher.
